# TGF-β1 in Vascular Wall Pathology: Unraveling Chronic Venous Insufficiency Pathophysiology

**DOI:** 10.3390/ijms18122534

**Published:** 2017-11-26

**Authors:** Pedro Serralheiro, Andreia Soares, Carlos M. Costa Almeida, Ignacio Verde

**Affiliations:** 1 Norfolk and Norwich University Hospital, Colney Ln, Norwich NR47UY, UK; andreiamsoares2@gmail.com; 2 Faculty of Health Sciences, CICS-UBI—Health Sciences Research Centre, University of Beira Interior, Av. Infante D. Henrique, 6201-506 Covilhã, Portugal; iverde@fcsaude.ubi.pt; 3 Department of General Surgery (C), Coimbra University Hospital Centre, Portugal; Faculty of Medicine, University of Coimbra, Praceta Prof. Mota Pinto, 3000-075 Coimbra, Portugal; c.m.costa.almeida@gmail.com

**Keywords:** chronic venous insufficiency, growth factors, signal transduction, TGF-β1, inflammation

## Abstract

Chronic venous insufficiency and varicose veins occur commonly in affluent countries and are a socioeconomic burden. However, there remains a relative lack of knowledge about venous pathophysiology. Various theories have been suggested, yet the molecular sequence of events is poorly understood. Transforming growth factor-beta one (TGF-β1) is a highly complex polypeptide with multifunctional properties that has an active role during embryonic development, in adult organ physiology and in the pathophysiology of major diseases, including cancer and various autoimmune, fibrotic and cardiovascular diseases. Therefore, an emphasis on understanding its signaling pathways (and possible disruptions) will be an essential requirement for a better comprehension and management of specific diseases. This review aims at shedding more light on venous pathophysiology by describing the TGF-β1 structure, function, activation and signaling, and providing an overview of how this growth factor and disturbances in its signaling pathway may contribute to specific pathological processes concerning the vessel wall which, in turn, may have a role in chronic venous insufficiency.

## 1. Introduction

The term chronic venous disorder (CVeD) includes the full spectrum of morphological and functional abnormalities of the venous system, from telangiectasia to venous ulcers. Functional abnormalities of the veins of the lower extremities producing edema, skin changes, or venous ulcers are clinically known as chronic venous insufficiency (CVI)—a term reserved for advanced CVeD [1].

Although it is extremely common, the exact prevalence of CVeD remains elusive. Reports of prevalence of CVI vary from <1% to 40% in females and from <1% to 17% in males [2]. Prevalence estimates for varicose veins are even higher, <1% to 73% in females and 2% to 56% in males [3,4]. Several factors, unrelated to actual differences in population frequency, account for some of the variation in the prevalence estimations: accuracy in application of diagnostic criteria, quality and availability of medical diagnostic and treatment resources, or variations in samples composition with respect to age, race, gender and geographic location [2,5]. CVI not only affects a significant proportion of the population, but also causes considerable morbidity and adversely impacts the quality of life of those affected. All of these factors have an influence on health-care budgets and public spending [6,7].

The recurrent nature of the disease, the socioeconomic burden and the ineffectiveness of current treatment modalities emphasize the need for more CVI-related research. Several theories about venous pathophysiology and varicosities genesis in the lower limbs (e.g., venous stasis theory, arteriovenous fistula theory, diffusion block theory) are outdated or have been refuted [8].

More recent hypotheses proposed that a dysfunctional venous system follows venous wall and valvular damage, which are triggered by venous hypertension and are the result of sterile inflammatory reactions [8,9,10,11,12,13]. However, the molecular sequence of events that lead to venous wall remodeling and structural weakness is yet poorly understood. It has been suggested that venous hypertension and/or wall hypoxia originates endothelial activation and expression of growth factors, adhesion and signaling molecules, which lead to leukocyte activation and migration [9,12,13,14]. This inflammatory process is responsible for the secretion of mediators that may trigger a local dysregulation of metalloproteinases/tissue inhibitors of metalloproteinases (MMP/TIMP) ratio that prompts abnormalities in extracellular matrix (ECM) structure, leading to decreased elasticity and increased distensibility of the venous wall [11,12,13,15,16,17]. Tissue remodeling is a complex process that is controlled by a great variety of factors, including transforming growth factor beta one (TGF-β1) [15,18,19].

TGF-β1 is a highly complex polypeptide with a key role in the regulation of cell function (including proliferation, differentiation, migration and apoptosis) and physiological processes (including embryonic development, angiogenesis and wound healing) [20,21]. Not surprisingly, disruptions in TGF-β1 signaling have been associated with a wide range of human diseases such as cancer and various autoimmune, fibrotic and cardiovascular diseases [20]. Therefore an emphasis on understanding its signaling pathways (and possible disruptions) may be of great importance in the comprehension and management of specific diseases.

Aimed at shedding more light on the processes involved in venous pathophysiology, this review will focus on describing TGF-β1 structure, function, activation and signaling, and on providing an overview of how TGF-β1 and disturbances in its signaling pathways may contribute to specific pathological processes in the vessel wall which, in turn, may have a role in CVI.

## 2. TGF-β1 Family and Function

TGF-β1 is a highly complex polypeptide that belongs to the superfamily TGF-β, which contains more than 30 structurally related polypeptide growth factors in mammals. In general, the family members are subdivided into two functional groups: (I) the TGF-β like group that includes TGF-β (1 to 3), activins, inhibins and some growth differentiation factors (GDF); (II) the bone morphogenetic proteins (BMP) like group comprising BMPs, most GDFs and anti-müllerian hormone (AMH) [21]. The TGF-β superfamily members share a conserved cysteine knot structure, are ubiquitously expressed in diverse tissues and function during the earliest stages of development and throughout the lifetime of humans [21]. Disturbances in TGF-β superfamily pathways, including either germ-line or somatic mutations or alterations in the expression of members of these signaling pathways, often result in several pathological conditions [22,23].

TGF-β1 is the most important isoform of the family in the cardiovascular system and is present in endothelial cells (EC), vascular smooth muscle cells (VSMC), myofibroblasts, macrophages and other hematopoietic cells [24].

Knockout studies of TGF-β1 signaling components in mice offered the first indication of their critical role in vascular development and function [20]. Both EC and their supporting cells (VSMC and pericytes) are needed to form a mature vascular network and TGF-β1 has been proposed not only to affect EC and VSMC (e.g., their proliferation, differentiation, migration), but also to regulate the interaction between them [25,26,27]. TGF-β1 is able to act as a promoting and an inhibitory factor of angiogenesis.

In addition to the angiogenic effect, TGF-β1 can induce a process called endothelial-to-mesenchymal transition (EndMT), by which EC lose apical-basal polarity and acquire a mesenchymal migratory phenotype [28]. EndMT is essential during embryonic development and tissue regeneration/wound healing, playing a role in pathological conditions like fibrosis or contributing to the generation of cancer-associated fibroblasts that are known to influence the tumor-microenvironment favorable for tumor cells [29].

TGF-β1 has also been shown to be a key regulator of ECM synthesis and remodeling. Specifically, it has the ability to induce the expression and deposition of ECM proteins, as well as to stimulate the production of protease inhibitors that prevent their enzymatic breakdown [4,19,30,31,32,33]. Abnormalities found on the structural matrix components (e.g., collagen, elastin) and the resultant increasing of ECM stiffness/loss of elasticity are common observations in cardiovascular [34] and venous diseases [35]. Therefore, the participation of TGF-β1 in the pathogenesis of vascular pathologies associated with matrix remodeling and fibrosis is not surprising [22].

The widespread expression profile of TGF-β receptors in all immune cell types suggests TGF-β1 participation in broad activities of the immune system [36]. According to Goumans et al., it delicately regulates the tolerogenic versus immunogenic arms of the immune system to balance adequate host defense while limiting collateral inflammatory tissue damage [26].

It is worth mentioning that this multifunctional growth factor is also known by its dual action. Indeed, the TGF-β1-elicited response is highly context-dependent throughout development and across different tissues. For instance, some authors reported that TGF-β1 protects bovine aortic EC from apoptosis [37], in contrast, others showed the opposite effect on porcine microvascular EC [37,38]. While at early stages of tissue repair TGF-β1, as a major orchestrator of the fibroproliferative response, stimulates the chemotaxis of repair cells, modulates immunity and inflammation, and induces matrix production; at later stages, it negatively regulates fibrosis through its strong antiproliferative and apoptotic effects on fibrotic cells [39,40]. Likewise, a dual role of TGF-β in the tumor microenvironment was described; it seems to prevent tumor growth and angiogenesis at early phases of tumor development, whereas it has pro-angiogenic and tumor promotion activities at late-stages of tumor progression [41,42]. Moreover, numerous in vitro studies also showed a dose-dependent (and timing-dependent) action of TGF-β1; for example, EC invasion and capillary lumen formation are inhibited by high concentrations of TGF-β1, whereas lower concentrations potentiate the effects of basic fibroblast growth factor and vascular endothelial growth factor induced invasion [43,44].

## 3. TGF-β1 Signaling Pathways: Major Components and Regulation

### 3.1. TGF-β1 Latent Complexes

**Figure 1:**
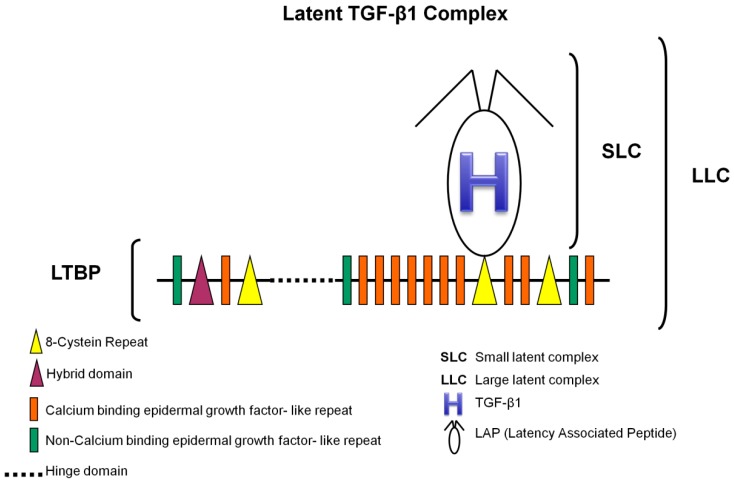
TGF-β1 large latent complex. LLC is composed of (I) a mature TGF-β1; (II) a latency associated peptide, which is a 75 kD homodimer with three side chains, two of which have the amino acid asparagine linked to mannose-6-phosphate oligosaccharides; and (III) a latent TGF-β binding protein of 125–160 kD, that contains 17 epidermal growth factor-like domains (14 of which are associated with calcium binding sequences) and four 70-amino-acid modules with eight cysteines each (adapted with permission from figure 1 in Rifkin DB. Latent transforming growth factor-beta [TGF-beta] binding proteins: Orchestrators of TGF-beta availability. *J. Biol. Chem.* **2005**, *280*, 7409–7412 [48]).

TGF-β1 (Figure 1) is synthesized as an inactive protein precursor (i.e., a pre-pro-protein) consisting of a signal peptide, an N-terminal prodomain and a C-terminal biologically active peptide [21,33,45]. Early on, it was realized that TGF-β1 is secreted from cells in a latent complex with its prodomain (latency-associated peptide or LAP) [32,46], indicating that during synthesis non-covalent interactions are formed between the prodomain and the mature domain, and a small latent complex (SLC) comes to existence. During the secretory process, the prodomain of TGF-β1 interacts covalently with a latent TGF-β-binding protein (LTBP) to form a large latent complex (LLC) which is then secreted into the ECM [4,32,33,47,48]. LTBP associates with TGF-β1 prodomain via signature 8-Cys region, which is unique to those proteins (Figure 1).

LTBP is required for secretion and correct folding of TGF-β1. The association with LTBP results in the storage of latent TGF-β1 in ECM structures rapidly after secretion [19,46,47,49]. It remains inactive in these structures (disulfide bond prevents it from binding to its receptors) until there is a proteolytic cleavage of LAP or a conformational change in LAP (induced by contractile forces)—critical events for protein activation [30,48,50].

In summary, latent complexes act to control TGF-β1 activity by sequestering it in the ECM or by mediating interactions with integrin receptors to release the mature peptide [50]. Thus, aberrant expression of the binding proteins can result in improper TGF-β1 signaling [23].

### 3.2. TGF-β Receptors and Smads

TGF-β1, as any other TGF-β family member, elicits its cellular effects by binding to different receptors. These are heteromeric complexes comprised of type I (activing-like kinase or ALK: ALK1, ALK2 and ALK5; ALK5 is also termed TGF-βR1) and type II (TGF-βR2) transmembrane serine/threonine kinase receptors. Type II receptors are constitutively active kinases capable of binding TGF-β1 alone, while type I receptors can only bind the ligand in cooperation with type II receptors. There are also type III receptors or coreceptors (betaglycan, also termed TGF-βR3, and endoglin), that bind to TGF-β1 and regulate this growth factor binding to its corresponding receptors, though they do not signal directly [51]. The type III receptors bind differing profiles of TGF-β family members. For instance, TGF-βR3, but not endoglin, can bind TGF-β2, an important distinction as TGF-β2 cannot otherwise bind to type II receptors. Thus, cells lacking TGF-βR3 are insensitive to TGF-β2 [52].

Through intracellular mediators, known as Smads, the TGF-β1 pathway can directly transduce extracellular cues from the cell-surface transmembrane receptors to the nucleus. This well conserved family (eight in mammals) can be divided into three functional classes: receptor-regulated Smads (R-Smads: Smad1, Smad2, Smad3, Smad5 and Smad9, which is mostly known as Smad8), common-mediator Smads (Co-Smad: Smad4) and inhibitory Smads (I-Smad: Smad6 and Smad7) [21,22]. All R-Smads have a C-terminal SSXS motif, within which the last two serines are directly phosphorylated by the type I receptor, while I-Smads lack the C-terminal SXS phosphorylation motif and thus act as inducible inhibitors (negative regulators) of the pathway [21,26,53].

### 3.3. TGF-β1 Signaling Pathways

Consistent with other TGF-β family members, TGF-β1 uses several intracellular signaling pathways to regulate a wide array of cellular functions. In addition to the canonical pathway (the Smad-mediated signaling pathway, which is ubiquitous and functions universally in all cell types examined) there are noncanonical pathways (the Smad-independent or non-Smad signaling pathways) [21].

**Figure 2:**
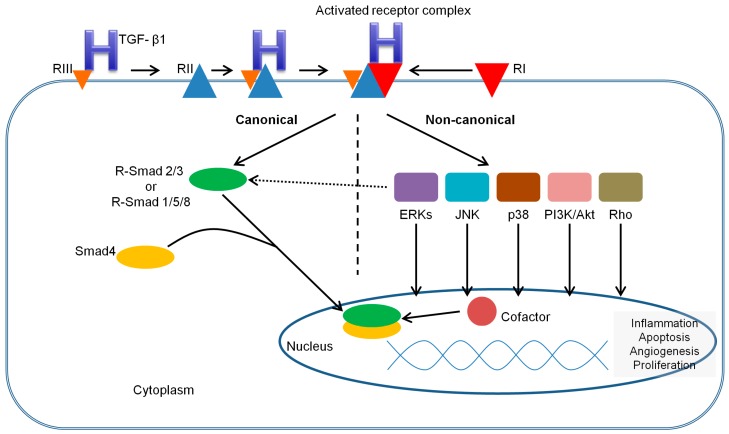
Smad-mediated TGF-β signaling system. Signal transduction by TGF-β1 is mediated via specific heteromeric complexes of type I and type II receptors. In most cells TGF-β1 interacts with TGF-βR2 (referred as RII) and ALK5 (referred as RI), but in EC it can also signal via ALK1 (also referred as RI). Coreceptors TGF-βR3 and endoglin (both referred as RIII) can facilitate TGF-βR2/ALK5 and TGF-βR2/ALK1 signaling. Activated receptor complex induces interaction and phosphorylation of R-Smad2/3 [26,53]. Upon phosphorylation, the two activated R-Smads form a heteromeric complex with Smad4. This complex is translocated into the nucleus, where it can interact with various transcription factors, coactivators or corepressors to modulate the expression of a multitude of genes, resulting in regulation of cellular responses (e.g., proliferation, differentiation, apoptosis).

Mechanisms of Smad-mediated TGF-β1 signaling are shown in Figure 2. Briefly, TGF-β1 initiates its signaling by binding to high-affinity cell surface receptors, type I and type II receptors. TGF-β1 binds to TGF-βR2, resulting in conformational changes that induces recruitment and complex formation with an appropriate type I receptor. Within the heterotetracomplex just formed, two type II receptors transphosphorylate two type I receptors in the glycine serine rich domain, activating their serine/threonine kinase activity [53,54]. In turn, the activated type I receptors mediate cellular effects through interaction and phosphorylation of R-Smads (ALK5 mediates the phosphorylation of R-Smad2/3, while ALK1/2 mediate the phosphorylation of R-Smad1/5/8) [26,53]. Upon phosphorylation, two activated R-Smads form a complex with Co-Smad, and this complex moves into the nucleus, where it combines with transcriptional activators and repressors, modulating target gene expression in a cell type-dependent manner [53,54]. The activation of R-Smads can be inhibited by I-Smads, which can compete for TGF-RI interaction, recruiting specific ubiquitin ligases or phosphatases to the activated receptor complex [26,53].

A number of noncanonical signaling cascades that operate in a context-dependent manner and contribute to cell-specific biological responses have also been elucidated (Figure 2). Among those are, for example, the mitogen-activated protein kinase (MAPK) pathways, including the extracellular signal-regulated kinases (Erks), c-Jun amino terminal kinase (JNK), p38 MAPK, as well as the IkB kinase (IKK), phosphatidylinositol-3 kinase (PI3K) and Akt, and Rho family GTPases [21,55]. These non-Smad receptor-activated transducers mediate signaling responses, either as stand-alone pathways or in conjunction with Smads, and control Smad activities [55]. While many function in concert with Smads to promote TGF-β1 responses (as in the case of Par6 phosphorylation and Smads during TGF-β1 induced epithelial-to-mesenchymal transition), activation of MAPK can result in phosphorylation of the Smad linker which can then inhibit Smad function by preventing nuclear accumulation of Smads or by promoting Smad degradation [55]. The complexity of TGF-β signaling pathway cross talk with other pathways has become increasingly apparent, as well as how subtle perturbations can result in pathological dysregulation [55].

### 3.4. Regulation of TGF-β1 Signaling

TGF-β1 signaling is tightly regulated at different levels, from the availability of the ligand, to the amount of receptors on the cell surface and the downstream transcription factors that will ultimately dictate the outcome of TGF-β1 signaling [56]. The regulation begins at the ligand level, during the proteolytic cleavage of LAP that will activate TGF-β1. This cleavage is induced by acidic environmental conditions or executed by extracellular proteases, including thrombospondin-1, plasmin, cathepsin D, MMP2 and MMP9, and furin convertase [32,33,48,57]. In the absence of proteolytic cleavage, upon mechanical stretch, αVβ6 integrin can activate TGF-β1, by binding to the RGD motif present in LAP and inducing the release of mature TGF-β1 from its latent complex [57]. Decorin, a small leucine-rich proteoglycan, seems to enhance TGF-β bioactivity in VSMC without change in total TGF-β protein, suggesting a mechanism of regulation involving post-translational control of TGF-β activation, active TGF-β availability or enhanced receptor signaling complex formation [58].

TGF-β1 signaling is also regulated at the level of its receptors, with FK506-binding protein 12 (FKBP12) that binds type I receptors (thus competing with type II receptors) to maintain them in an inactive conformation [59]. Once activated, TGF-β receptors are internalized through two major endocytic pathways, clathrin-mediated endocytosis and lipid raft/caveolae-mediated endocytosis. Internalization of TGF-β receptors through clathrin-dependent endocytosis enhances Smad-mediated TGF-β1 signaling, whereas caveolin-mediated endocytosis promotes the ubiquitination and degradation of the receptors and thus signaling turnoff [60]. Several regulators of TGF-β receptor activity and stability have been identified, such as Casitas B-lineage lymphoma (c-Cbl) [61], the protein that interacts with C kinase 1 (PICK1) [62] or Dapper2 [63]. In addition, TGF-β coreceptors regulate the cell surface localization, internalization and signaling of their respective signaling receptors through interactions with the scaffolding proteins like GAIP-interacting protein C-terminus (GIPC) and β-arrestin2 [64].

At the Smad level, Smad2/3 binding to FYVE domain proteins (e.g., ZFYVE9, better known as hSARA) retains them in the cytoplasm awaiting activation by their respective type I receptor [65]. Interaction between phosphorylated Smads and Smad4 is also regulated, as ErbB2/Her2-interacting protein (Erbin) sequesters Smad2/3 in the cytoplasm away from Smad4, thus preventing TGF-β signaling [66]. In addition, several phosphatases, such as PPM1A or small C-terminal phosphatases, dephosphorylate Smads at this site either in the cytoplasm or in the nucleus to terminate Smad activity [67].

TGF-β1 signaling regulation proceeds into the nucleus, where Smad complexes target specific promoters to regulate gene expression patterns. Given the low-affinity Smad/DNA binding, Smads are generally dependent on direct interaction with specific high-affinity DNA binding proteins (FOXH1 protein is just one of a growing list [60]) for recruitment to appropriate target genes [21]. Upon binding to DNA in partnership with specific transcription factors, Smads recruit coregulators (i.e., coactivators such as basic chromatin remodeling complexes and histone-modifying acetyltransferases or corepressors such as histone deacetylases) to promote or inhibit initiation of transcription [21,60].

Once the pathway is activated, a number of feedback mechanisms are turned on to regulate the duration and strength of the signal. For example, I-Smads, whose production is directly increased by TGF-β signaling, act as negative regulators of the pathway by forming stable complexes with activated type I receptors and thereby blocking the phosphorylation of R-Smads [68]. They also recruit WW-HECT-type E3 ubiquitin ligases (viz. Smad ubiquitin-related factor-1 or Smurf1, Smurf2, NEDD4-2 and WWP1) to type I receptors, which are essential for ubiquitination and degradation of the receptor complex, as well as the termination of the process [68]. Besides, TGF-β signaling pathways also stimulate the production of secreted proteins (e.g., protein acidic rich in cysteine or SPARC, cystatin C, fibulin-5) that function to both mediate and regulate signaling [69,70,71]. In the nucleous, Smad7 directly binds to DNA and represses TGF-β signaling by interfering with the functional R-Smad/Smad4-DNA complex [56].

Given the complexity of TGF-β1 signaling and its regulation, it is not a surprise that aberrant expressions of the signaling components and signaling pathway dysregulation often result in human disease, as explained below.

## 4. The Role of TGF-β1 Signaling Pathways in Vessel Wall Pathological Processes

### 4.1. TGF-β1 and the Vascular Wall Shear Stress

Mechanical forces imposed by pulsatile flow of blood, which include frictional wall shear stress, circumferential distention and blood pressure, play an important role in maintaining vessel structure and function [72,73]. EC lining the vasculature are continuously exposed to shear stress, leading to reorganization of its cytoskeleton, to morphological alterations and to the production of a variety of substances that act on EC themselves and on surrounding cells (e.g., VSMC) [74]. Failure to adapt to shear stress results in endothelial damage, which may lead to generation of atherosclerotic plaques or abnormal vessel repair [73,75,76].

Several studies were able to link TGF-β1 production and vascular remodeling induced by shear stress. It was demonstrated that human arterial and venous VSMC exposed to chronic cyclical mechanical strain responded, in a “dose-dependent” fashion, with an increase of TGF-β1 mRNA expression and matrix accumulation [77]. According to the authors’ proposal, this would most likely represent the main biological mechanism whereby hypertension promotes cardiovascular matrix accumulation [77].

Other authors could confirm that low shear stress (which occurs preferentially at vessel branch points, bifurcations and regions of high curvature) was a pathological inducer for vascular remodeling by upregulated migration and proliferation of EC and VSMC [76]. An increased paracrine secretion of platelet-derived growth factor-BB (PDGF-BB) and TGF-β1 from EC induced by low shear stress was found, as well as the activation of ERK 1/2 and affected expressions of LOX and lamin A—processes that were suggested as having a possible role in the effects of PDGF-BB and/or TGF-β1 on cellular migration and proliferation [76].

Furthermore, observations on vein graft remodeling have identified hemodynamic forces (wall shear and tensile stresses) as the primary stimuli that induce active reorganization of the graft wall, and a role for TGF-β1 in this event. Evidence supporting the concept that increased wall stress after vein graft implantation induces the recruitment of adventitial fibroblasts, mediated by a connective tissue growth factor (CTGF, also known as CCN2) and TGF-β1, and the conversion to a myofibroblast phenotype has been provided [78,79]. This adventitial adaptation, despite being important in the maintenance of wall stability (in response to an increased mechanical load), limits the early outward remodeling of the vein conduit and may have a detrimental effect on maintaining the long-term vein graft patency [78]. Recent findings (such as suppression of TIMP1, enhanced expression of TGF-β1 and BMP-2 mRNA or upregulation of microRNA-138/200b/200c) were consistent with the previous results and suggested a role of arterial-like wall strain in the activation of pro-pathological pathways, resulting in adventitial vessel growth, activation of vasa vasorum cells and upregulation of specific gene products associated to vascular remodeling and inflammation [75].

The potential role of TGF-β signaling in mediating the protective effects of physiological shear stress on EC was also studied. Interestingly, the results revealed that shear stress induced TGF-β3 signaling and a subsequent activation of Kruppel-like factor 2 and nitric oxide (NO), indicating that TGF-β3 (but not TGF-β1) has a critical role in the maintenance of endothelial homeostasis in a hemodynamic environment [72].

### 4.2. TGF-β1 and Vascular Wall Fibrosis

**Figure 3:**
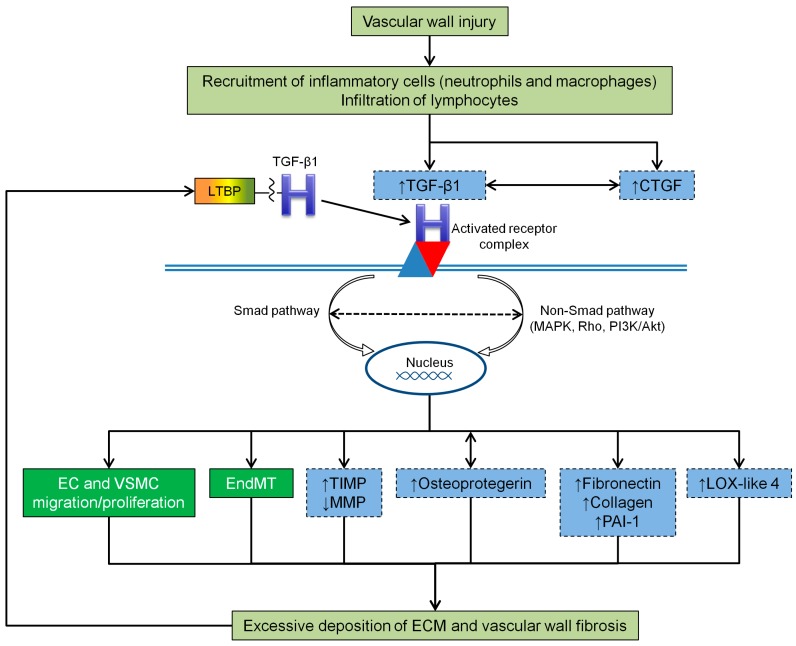
Mechanisms linking vascular wall injury, inflammation and vascular remodeling. TGF-β1 (transforming growth factor-beta one), LTBP (latent TGF-β-binding protein), CTGF (connective tissue growth factor), EC (endothelial cells), VSMC (vascular smooth muscle cells), EndMT (endothelial-to-mesenchymal transition), TIMP (tissue inhibitors of metalloproteinases), MMP (metalloproteinases), PAI-1 (plasminogen activator inhibitor one), LOX-like 4 (lysyl oxidase-like four), ECM (extracellular matrix).

In physiological conditions, fibrosis is a process of normal wound healing and repair, activated in response to injury, to maintain the original tissue architecture and its functional integrity. It involves a complex multistage process with recruitment of inflammatory cells, release of fibrogenic cytokines and growth factors (such as TGF-β1) and activation of collagen-producing cells [80]. However, prolonged chronic stimuli lead to a long term activation of myofibroblasts (a specialized type of fibroblasts that are normally activated during wound healing) [81], which in turn may result in excessive and abnormal deposition of ECM and fibrosis (Figure 3). If the build-up of ECM occurs in organs (e.g., lungs, liver, kidneys and skin) it can interfere with their function and, if it continues unabated, leads to organ failure [23].

As mentioned before, TGF-β is a key regulator of ECM, thus its excessive signaling has long been implicated in the pathogenesis of vascular fibrosis and other fibrosis-related diseases. Moreover, it acts as a mediator of vascular fibrosis induced by several agents involved in vascular diseases (e.g., mechanical stress, angiotensin II, high glucose, advanced glycation products) [22,72,82,83]. The heat shock protein 70 (HSP70), whose primary function is to repair denatured proteins through folding/unfolding steps and thus achieve correct functional configuration, is another example of an agent believed to stimulate TGFβ1-induced ECM accumulation and to contribute to the inflammation and fibrosis present in fibrosis-related diseases [84].

Several genes encoding ECM proteins that are known to be important in driving fibrosis are directly regulated by TGF-β signaling, through Smads [85,86,87] as well as with the involvement of MAPKs, Rho family members and reactive oxygen species [22]. TGF-β1, at low concentrations, increases the synthesis of ECM proteins, such as fibronectin, collagens and plasminogen activator inhibitor one (PAI-1) in VSMC, EC and fibroblasts [88,89,90] (Figure 3). The synthesis of fibronectin by VSMC, via the TGF-β1/Smad3 signaling pathway, leads to the deposition of ECM in the neointima [91]. Other authors have shown that in a high-phosphate environment, the upregulation of fibronectin in cultured VSMC takes place via TGF-β1 production [92]. The reduction of type I and III collagen secretion by VSMC is induced through TGF-β1/Smad3 signaling pathway inhibition [93]. Expression levels of PAI-1, the major physiological regulator of the plasmin-based pericellular proteolytic cascade, are also linked to TGFβ1-induced neointimal expansion [89], although the actual role of PAI-1 in the development of a VSMC-rich neointima is likely to be complex [94,95].

A member of the lysyl oxidase family of matrix-remodeling enzymes, lysyl oxidase-like four (LOX-like 4), was also identified as a direct target of TGF-β1 in EC and evidence that LOX-like 4 was extracellularly secreted and significantly contributed to ECM deposition was provided [96]. Such results suggest that TGFβ1-dependent expression of LOX-like 4 might have pathophysiological implications in vascular processes associated with matrix remodeling and fibrosis [96] (Figure 3).

It has been suggested that TGF-β1 and CTGF synergize to promote chronic fibrosis [22] (Figure 3). CTGF is expressed predominantly in embryonic and adult vasculature, regulates various biological processes associated with fibrogenesis (including cellular adhesion, proliferation, differentiation, apoptosis, ECM production and angiogenesis) [97,98,99] and was found upregulated in a variety of fibrotic disorders [99]. The enhancement of TGF-β1 activity via CTGF may occur through the following mechanisms: CTGF not only increases the affinity between TGF-β1 and its receptors (by binding directly to the former), but also leads to Smad7 transcriptional suppression (via induction of the transcription factor TIEG) [22]. Evidence from in vitro studies indicated that CTGF might act as a downstream target of TGF-β1, as treatment with exogenous TGF-β or exogenous CTGF significantly upregulated the TGF-β/CTGF pathway and increased the expression levels of ECM components [100]. In addition, studies with animal vein bypass grafts showed that an enhanced signaling via TGF-β/CTGF, coupled with the reduced MMP2 and MMP9 activity, promotes progressive ECM accumulation and neointimal fibrosis during late neointimal expansion in vein grafts [101].

Similarly, it seems that osteoprotegerin promotes fibrogenesis in VSMC via a TGF-β1 autocrine loop [102] (Figure 3). This member of the tumor necrosis factor receptor family was found increased in VSMC of the vascular media in response to TGF-β1, in a vicious cycle that results in the auto-induction of TGF-β1, and was associated with upregulation of fibrogenesis [102].

The inhibition of ECM degradation (leading to excessive ECM accumulation) can also be reinforced by TGF-β1 action on TIMP expression, which once stimulated helps to decrease collagenase production and activity [22,88] (Figure 3).

An additional contributor to fibrosis is the previously mentioned EndMT process (Figure 3), which is associated with increased expression of fibroblast-associated proteins [23], such as alpha smooth muscle actin (α-SMA) [78]. Indeed, TGF-β1 can induce the transformation of fibroblasts into α-SMA expressing myofibroblasts and promote the secretion of ECM [78,79,93]. EndMT elaboration can further exacerbate fibrosis, as observed, for example, in renal fibrosis [103] or in arterial restenosis following surgical trauma [104]. Studies addressing vein graft remodeling collected data that not only establishes EndMT as an important mechanism underlying neointimal formation in vein grafts, but also identifies a pivotal role for TGFβ1/Smad2/3-Slug signaling pathway in regulating vein graft EndMT [105]. Moreover, in the presence of elevated levels of Smad3, TGF-β1 can promote a proliferative and/or migratory phenotype on VSMC, that can aggravate neointimal formation following vascular surgery [106,107]. More recently, it has been proposed that TGF-β1/Smad signaling and MMP14 act to recruit mesenchymal stem cells which differentiate to VSMC and mesenchymal-like cells that participate in vascular repair and remodeling [108].

Intriguingly, these well-documented effects of TGF-β1 activity on ECM remodeling (and its consequent mechanical property changes) seem to be part of a vicious cycle that drive cell fibrogenesis, since ECM straining and stiffening have an important impact on the activity and availability of this pro-fibrotic growth factor [50]. According to the author’s hypothesis, during the gradual remodeling process, ECM will eventually reach an organization degree that lowers the threshold for TGF-β1 activation, by increasing the mechanical resistance to cell pulling, and the available TGF-β1 will then contribute to the activation of fibroblasts into contractile myofibroblasts, just when further remodeling of the strained and stiffened ECM requires stronger cell forces [50].

### 4.3. TGF-β1 and Venous Wall Abnormal Morphology and Functioning

Healthy veins of the lower extremities are equipped with efficient walls, contractile VSMC and competent valves in order to withstand the high hydrostatic venous pressure in the lower limbs and allow unidirectional movement of deoxygenated blood toward the heart [17,18]. In contrast, varicose veins (a common clinical manifestation among patients with CVI [3]) appear to be dilated, elongated, tortuous and often show incompetent venous valves and a measurable venous reflux [8,18]. Moreover, structural and histological evidence suggest that varicose veins have both hypertrophic (with abnormal VSMC shape and orientation and ECM accumulation) and atrophic (with ECM degradation and an increase in inflammatory cell infiltration) regions [12,109,110] and no clear boundaries among vascular layers—irregular distribution of collagen bundles or thickened and fragmented elastic fibers may be found throughout the vein wall [111,112], making it difficult to distinguish between tunica intima, media and adventitia.

The primary reason for this extensive ECM remodeling and structural weakness of the vein wall has still not been clearly explained, but numerous factors, including TGF-β1, seem to be implicated not only in the pathogenesis of varicose veins, but also in numerous complications associated with varicose veins (e.g., thrombophlebitis, lipodermatosclerosis, venous ulcers) [13,113,114].

Data regarding TGF-β1 expression/activity in patients with CVI remains inconclusive (Table 1). Some studies reported unchanged TGF-β1 levels in cell cultures from varicose veins [115] and comparable amounts of TGF-β1 mRNA levels [113] or TGF-β1 active form in normal and varicose veins [109]. By contrast, others demonstrated increased TGF-β1 mRNA levels, protein expression, immunoreactivity and total content in the walls of varicose veins [109,116,117], or decreased protein expression of TGF-β1 latent [19,113] and active [113] forms in varicose veins when age-related differences were controlled.

Furthermore, as tissue responsiveness to TGF-β1 depends on several factors (e.g., TGF-β signaling components availability) rather than on its activation/availability alone, a few studies [113] evaluated mRNA expression and protein expression of TGF-βR2/3 and Smad2/3 in varicose vein walls and achieved, once more, opposite results. While Kowaleski et al. observed increased protein expression of TGF-βR2 and Smad2/3 in varicose veins (from patients assigned to class C2 of CEAP classification system), we found [119] decreased TGF-βR2/3 gene expression and TGF-βR2 immunoreactivity in varicose veins (from patients assigned to different classes: C2 to C6). Interestingly, based on a review of several studies reporting dermal fibroblast responsiveness to TGF-β1 in venous ulcers [120], it was suggested that slower proliferative response of fibroblasts throughout CVeD progression was associated with a decrease in TGF-βR2 expression (and consequent abnormalities in the downstream signaling pathway, i.e., failure of ulcer fibroblasts to phosphorylate Smad2/3 and p42/44 MAPK [121]), ultimately leading to senescence and poor ulcer healing.

The conflicting results regarding TGF-β1 (and its signaling components) expression/activity may be partially explained by important methodological differences: for example (no) use of effective control samples (i.e., control and varicose veins were harvested from patients with CVI), when some argue that CVeD is a generalized disorder in the venous system [18,112]; (no) control of individual differences between control and experimental groups, when there is evidence that aging induces dysregulation of TGF-β1 [19]; (no) distinction between hypertrophic and atrophic varicose segments, when considerable heterogeneity regarding cellular and matrix components presence was already observed [12,109]; (no) separation of specimens based on anatomic harvest site, when vein source and location seem to be a factor of variability [122]. However, one can reasonably argue that these results also suggest that TGF-β1 secretion, activation and/or cell signaling could be impaired and this may be a central pathological mechanism in CVeD.

**Figure 4:**
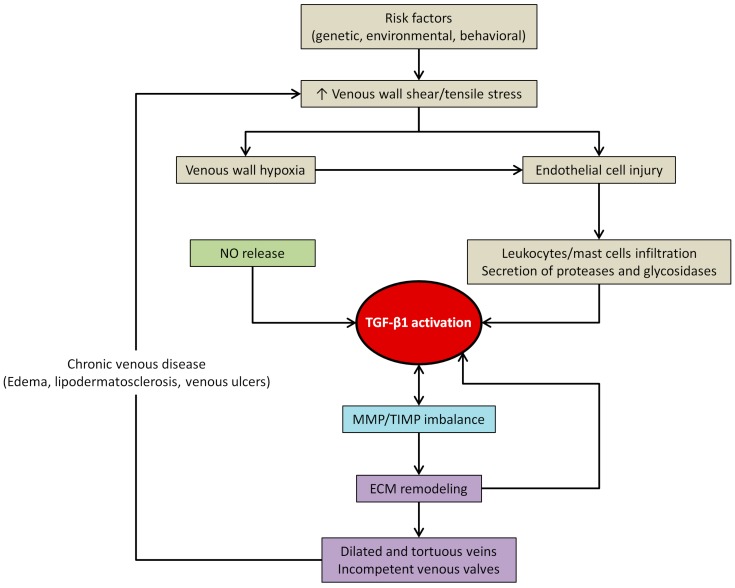
Schematic representation of chronic venous disease pathophysiology. NO (nitric oxide), TGF-β1 (transforming growth factor-beta one), MMP (metalloproteinases), TIMP (tissue inhibitors of metalloproteinases), ECM (extracellular matrix).

As explained earlier, hemodynamic forces (wall shear and tensile stresses) and inflammation are among the potential factors that could modulate the expression/activity of TGF-β1 in the vascular wall. An increase in venous hydrostatic pressure in the lower extremities (caused by certain genetic, environmental and behavioral risk factors) could lead to EC injury, increased permeability, activation of adhesion molecules and leukocyte infiltration—collectively these factors could contribute to vein wall inflammation [18,123,124] (Figure 4). In response to inflammation or injury, the active form of TGF-β1 can be released by a variety of mechanisms, including enzymes such as proteases and glycosidases secreted by leukocytes and mast cells [4,19,33]. Enhanced infiltration of mast cells was noted in varicose vein walls (particularly from elderly subjects) [19,125], suggesting that degranulation of mast cells may release enzymes into the ECM (e.g., tryptase, hydrolases, oxidative enzymes, carboxypeptidases) and contribute to TGF-β1 maturation or activation, as well as to venous wall diminished structural integrity [19] (e.g., mast cell tryptase catalyzes the degradation of different matrix components such as type IV collagen, elastin, fibronectin and extracellular proteoglycans [19,126]). Similarly, while studying the relationship between NO production (an important cellular-signaling molecule that regulates vascular tone and has diverse pathophysiologic functions such as inhibition of platelet adhesion/aggregation, mediation of the inflammatory cascade, among others) and TGF-β1 expression in varicose veins, it was found that TGF-β1 overexpression was correlated with overproduction of inducible NO synthase and with monocyte/macrophage infiltration in both tortuous and nontortuous varicose veins [117]. It was then speculated that NO released by the upregulation of NO synthase enhances the production of TGF-β1 in varicose veins, which in turn may be related to dysregulated cell cycle (e.g., decreased apoptosis) and to progressive hypertrophy of the vein wall [117] (Figure 4).

Due to TGF-β1 broad influence on ECM remodeling, vein wall imbalance of elastin and collagen content and the growth factor expression/activity (mostly in association with MMP/TIMP ratio changes) have also been an object of study. Depletion of vein elastic components is a common observation in the venous wall of elderly and CVI patients (particularly, during the disease later stages) [116,127] that was related to decreased elastin synthesis and increased elastase activity, which in turn could be a consequence of elastic tissue modulators (such as LTBP and TGF-β1) attempt to stabilize elastin expression in regions of extensive injury [116]. In addition, some authors suggested that abnormalities in ECM structure leading to decreased elasticity and increased distensibility of the venous wall result from an imbalance between MMP (endopeptidases involved in the degradation of several ECM proteins) and TIMP (their endogenous inhibitors) [12,15,16,18,112,128], which are regulated at both transcriptional and post-transcriptional levels by various cytokines or growth factors, including TGF-β1 [15,118] (Figure 4). In reverse, MMP upregulation may also promote growth factor release (by cleaving growth factor-binding proteins) and this may partly explain the VSMC hypertrophy observed in some parts of the hypertrophic regions of varicose veins [18].

Changes in TGF-β1 expression/activity have also been associated with CVeD progression and with numerous CVI-related complications. Several studies concerning advanced forms of dermal pathology in CVI patients (i.e., dermal fibrosis and venous ulcers) have identified CVI dermal fibroblasts as an important target for leukocyte-derived TGF-β1, and established a link between CVI progression and increased tissue levels of TGF-β1, MMP2 activity and decreased TGF-β1 induced mitogenic responses of fibroblasts [4,15,120,129,130]. Moreover, two other studies on CVI induced dermal changes suggested a connection between TGF-β1 signaling and cellular senescence [130,131]. It was also proposed that a molecular mechanism in which the persistent venous hypertension (via pressure mediated mechanotransduction) could stimulate Ras production, which in turn would activate MAPK/Erks, and hence inhibit TGF-β1 regulated matrix contraction (resulting in prolonged wound healing) [130]. Other authors, after observing differences in TGF-β1 expression/activity (as well as in some of its signaling components, such as TGF-βR2 and Smad2/3) among control veins, varicose veins and varicose veins complicated by thrombophlebitis, proposed that thrombophlebitis accelerates TGF-β1 activation and its signaling cascade in the varicose vein wall, which may have a role in the local inflammatory process [113].

## 5. Concluding Remarks

TGF-β1 plays a key role in many biological and pathological processes, some of which have an impact on cardiovascular homeostasis. Considerable progress has been made over the past several years in the understanding of biomechanical and structural aspects of this growth factor, including its activation and intracellular signaling (canonical and noncanonical) pathways.

Despite its high prevalence and detrimental socioeconomic impact, CVeD is far from being completely understood due to major reasons: a multifactorial aetiology and a great difficulty to identify the primary stimulus and map the sequence of pathological events (by the time CVI symptoms present clinically, a vicious cycle of pathological events is already in motion). It has become widely accepted that vein wall changes and valvular dysfunction (appointed as primary events) are triggered by blood stasis and venous hypertension and are the result of sterile inflammatory reactions. All the main mechanisms postulated to contribute to the disease onset and development (i.e., shift in hemodynamic forces, wall inflammation, ECM degradation/deposition, venous tone alteration) seem to affect or be affected by TGF-β1 expression and activity, which can be dysregulated.

While valuable research efforts have been made to understand the bimolecular mechanisms of severe complication of CVI, such as chronic venous leg ulcers, there is still much to understand about TGF-β1 role in the pathophysiology of both early and late stages of CVeD. To date, there are only a few studies regarding TGF-β1 expression/activity in CVeD patients, with a significant number of them reaching opposite results. The field requires not only more studies, but mostly methodological uniformity (e.g., use of effective control specimens, criteria for specimen anatomic harvest sites). Given the great number of components and regulation factors involved in TGF-β1 signaling, future studies should focus on the expression/activity of molecules beyond TGF-β1 itself (e.g., LTBP isoforms, TGF-β receptors, Smads, MAPKs, integrins). They should also take into account CVeD progression, as these molecules expression/activity may evolve throughout the disease stages. An example would be studying a few major TGF-β1 signaling components in segments of healthy and varicose veins, grouped by different disease stages and specific anatomical regions, to better understand how differences, if any, in these molecules expression/activity may explain vein wall structural evolution throughout CVeD progression.

Among CVeD treatment modalities, the outlook for pharmacotherapy targeting the TGF-β1 signaling pathway seems promising, yet challenging—current drugs targeting TGF-β1 and its signaling components are not selective for pathological signaling pathways and require careful side effects monitoring [20]. It is paramount that the development of further studies should be able to dissect TGF-β1 mechanisms of action as well as to identify new cell type regulators (that could be safely used for anti-TGFβ signaling therapy), in order to improve effectiveness in CVeD prophylaxis and treatment.

## Figures and Tables

**Table 1:** Studies regarding TGF-β1 expression and/or activity in veins.

Reference	Year	Specimen	TGF-β Expression and/or Activity in Veins
Badier-Commander C. [109]	2001	Varicose and non-varicose great saphenous vein segments	Increased total amount of TGF-β1 in varicose segments, but identical amount of active TGF-β1
Bujan J. [116]	2003	Varicose and non-varicose great saphenous vein segments	Increased TGF-β expression and decreased elastin synthesis in injured regions of varicose segments
Sansilvestri-Morel P. [115]	2005	Vascular smooth muscle cells (VSMC) from varicose and non-varicose great saphenous vein segments	Unchanged TGF-β1 levels
Jacob T. [117]	2005	Varicose and non-varicose great saphenous and tributaries vein segments	Increased TGF-β1 expression in varicose segments
Pascual G. [19]	2007	Varicose and non-varicose great saphenous vein segments from older subjects	Reduced latent TGF-β1 expression in varicose segments
Kowalewski R. [113]	2010	Varicose and non-varicose great saphenous vein segments	Unchanged TGF-β1 mRNA levels and decreased latent and active TGF-β1 expression in varicose segments
Serralheiro P. [118]	2017	Cultured healthy great saphenous veins	TGF-β1 increased mRNA levels of MMP9, MMP12, TIMP1 and TIMP2
